# Cell Sorting Using Electrokinetic Deterministic Lateral Displacement

**DOI:** 10.3390/mi12010030

**Published:** 2020-12-30

**Authors:** Bao D. Ho, Jason P. Beech, Jonas O. Tegenfeldt

**Affiliations:** Division of Solid State Physics and NanoLund, Physics Department, Lund University, PO Box 118, 22100 Lund, Sweden; bao.hodang@gmail.com (B.D.H.); jason.beech@ftf.lth.se (J.P.B.)

**Keywords:** electrokinetic deterministic lateral displacement, charge-based separation, dielectrophoresis

## Abstract

We show that by combining deterministic lateral displacement (DLD) with electrokinetics, it is possible to sort cells based on differences in their membrane and/or internal structures. Using heat to deactivate cells, which change their viability and structure, we then demonstrate sorting of a mixture of viable and non-viable cells for two different cell types. For *Escherichia coli,* the size change due to deactivation is insufficient to allow size-based DLD separation. Our method instead leverages the considerable change in zeta potential to achieve separation at low frequency. Conversely, for *Saccharomyces cerevisiae* (Baker’s yeast) the heat treatment does not result in any significant change of zeta potential. Instead, we perform the sorting at higher frequency and utilize what we believe is a change in dielectrophoretic mobility for the separation. We expect our work to form a basis for the development of simple, low-cost, continuous label-free methods that can separate cells and bioparticles based on their intrinsic properties.

## 1. Introduction

While standard cell sorting schemes rely on labelling and molecular recognition events, cells have important properties for which there exist no labels. This has driven the development of microfluidics-based label-free techniques that exploit cells’ intrinsic physical properties for fractionation. The targeted properties can be size [1,2,3,4,5], shape [6,7], compressibility [4], dielectric properties [8], or any other physical characteristic, which can be used as a handle to apply a separating force.

**Figure 1:**
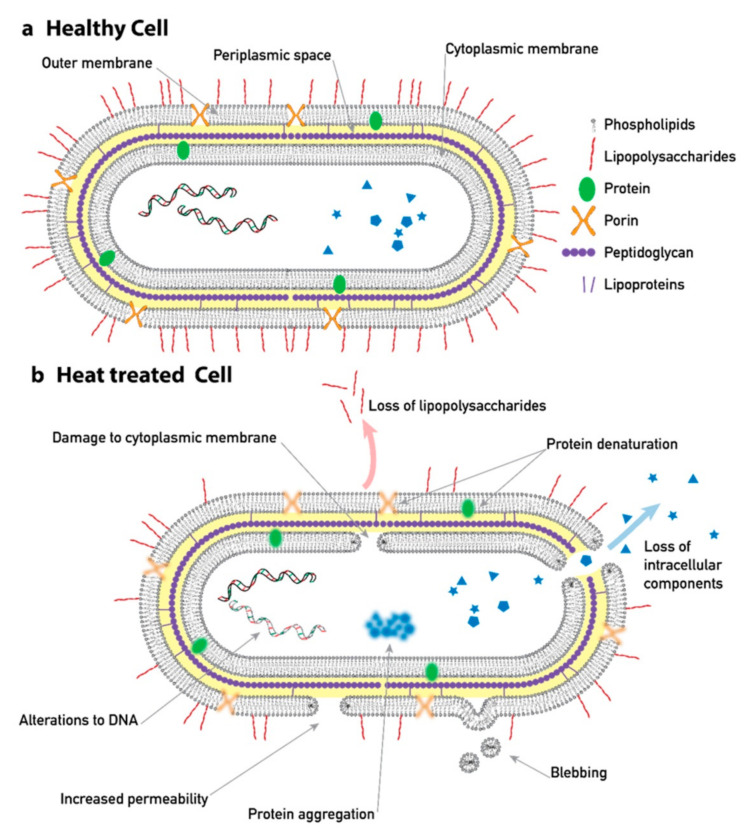
An overview of typical structural changes that occur as a consequence of heat treatment. Here, the cell is a gram-negative bacteria, but similar mechanisms occur in other cell types. Structural changes of a cell are reflected in its dielectric and electrokinetic properties due to, e.g., changes in the conductivity of the membrane, the charge of membrane, and aggregation of protein. Schematics are based on descriptions of heat-induced changes in [9,15].

Dielectric and electrokinetic properties of the cells can be strongly affected by changes of the structure of the cells [9] (see Figure 1). Dielectrophoresis (DEP) is a well-established technique that can be used to target these types of changes. It is based on the movement of particles along an electric field gradient due to their dielectric properties [10,11]. The sign of the force depends on the difference between the polarizability of the particle and its surrounding medium. The particles may thus experience a force towards higher electric fields (positive DEP) or towards lower electric fields (negative DEP). Adjusting the frequency of the applied electric field, the force can be tuned and made sensitive to the desired types of changes of the particles of interest. One common way to change the structure of cells is heat treatment. It is known to impart structural changes to the membrane and cell wall as well as internal components affecting the viability of yeast [12,13] and bacteria [14,15], with important implications for the food industry. DEP has, therefore, been found useful to characterize heat-treated cells with respect to viability. Pohl and Hawk published a pioneering paper in 1966 showing the ability to separate live yeast cells from stained dead cells using DEP with a simple device consisting of two macroelectrodes inside a PMMA (polymethyl methacrylate) fluid chamber [16]. Markx et al. showed similar results but with more sophisticated devices featuring castellated microelectrodes deposited at the bottom of a microfluidic chamber [17,18]. By adjusting the frequency of the AC voltage applied between adjacent electrodes and switching the flow and voltages, the authors implemented a trap and release separation scheme. Trapping of viable and non-viable *Escherichia coli* bacteria using DEP in insulating array structures has also been reported [19]. Here, electroosmosis was used for flow, and live/dead bacteria were trapped at different positions in the insulating array using DC-DEP and a difference in their DEP mobility. Not only DEP but also other types of electrokinetic forces can be leveraged for particle sorting. We showed recently, how zeta potential can be used as a basis for sorting of particles of different surface charge [20].

In this work, we will use untreated and heat-treated *Escherichia coli* and *Saccharomyces cerevisiae* as model systems for cells with different internal structure to demonstrate the capabilities of a combination of electrokinetics and deterministic lateral displacement (DLD) to sort cells.

DLD is a powerful mechanism for sorting particles based on size [2]. Among the strengths of DLD are that it is continuous, making it suitable for integration with other methods (see the CTC-iChip [21]), it has high size resolution [2], and has been demonstrated to work for a wide range of particle sizes, from millimeters [22] to nanometers [23]. As a result, DLD has been employed in various microfluidic devices to sort a wide range of cells and bioparticles, i.e., white blood cells from red blood cells and plasma [24,25,26], circulating tumor cells from blood [27,28], and trypanosomes from blood [29,30].

**Figure 2:**
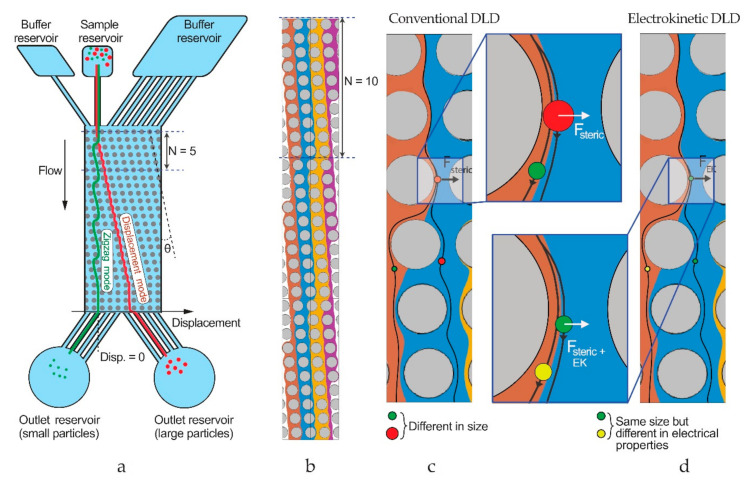
Working principle of deterministic lateral displacement (DLD). (**a**) An array of pillars tilted with an angle splits a mixture of particles into different trajectories based on size. At the end of the array, the different-sized particles (green and red) exit at different lateral positions and are collected into different outlet reservoirs. (**b**) Simulated flow streams within a DLD array, showing zigzagging patterns. (**c**) Mechanism of sorting: steric interactions between particles and posts cause particles to change flow streams in a size-dependent manner that leads to separation. (**d**) In an electrokinetic DLD device, electrokinetic forces act on the particles in addition to the steric forces, modifying separations.

The basic mechanism of DLD is described in Figure 2. Particles move through the array in one out of two basic modes governed by steric interactions with the posts in the array, small particles following the flow in the zigzag mode and large particles deviated laterally according to the array geometry in the displacement mode. The threshold dia-meter between the two modes is called the critical diameter, which can be estimated by an empirical formula presented by Davis [31]:

(1) $$D_{C}=1.4GN^{-0.48}.$$

Here, *G* is the gap between adjacent pillars and *N* is the period of the pillar array, the number of rows along the flow direction after which the array repeats itself.

Through additional force interactions between the posts and the particles, the device can be made more versatile. Electrostatic interactions between particles and posts have been exploited as a means to bias the DLD and make it sensitive to the charge of particles [32]. Another approach is to add electric fields to the device so that electrokinetic forces affect the sorting, opening up for tunability and more specific sorting [33]. The approach is known as electrokinetic DLD (eDLD), and we will apply it here as described recently [20].

## 2. Materials and Methods

### 2.1. Devices and Experimental Setup

**Figure 3:**
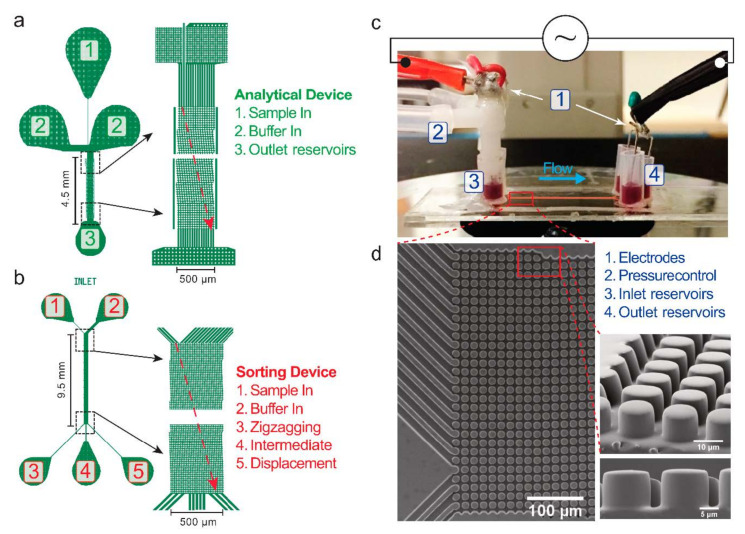
Details of the devices and the experimental setup. (**a**,**b**) Two types of designs used. (**c**) Side view of a device and the experimental setup, with pressure and electrical connections. (**d**) SEM images of the DLD array of Sorting device #1, which is representative for all devices used in this work.

The DLD devices were cast in polydimethylsiloxane (PDMS) using replica molding [34]. Details can be found in Table S1, Supplementary Information. We have used two types of designs: Analytical devices (Figure 3a and Table 1) and Sorting devices (Figure 3b and Table 2). In an Analytical device, only a narrow, single stream of sample is allowed to enter the DLD array and is buffered on two sides. The particles in the sample are separated as they travel along the array before entering a single outlet reservoir. The lateral positions of the particles are analyzed at the observation windows at the beginning and at the end of the array. In the second type of device, the Sorting device, the input sample stream is much wider to increase throughput. At the outlet side, there are several output reservoirs collecting particles that are displaced by different amounts and that can be counted externally.

The setup of an experiment is shown in Figure 3c. The device was placed on the stage of an inverted microscope (Nikon Eclipse TE2000-U, Nikon Corporation, Tokyo, Japan). At inlet reservoirs, an overpressure (1–100 mBar) was applied via a pressure controller (MFCS-4C, Fluigent, Paris, France). Together with the pressure, a voltage was applied between inlet and outlet reservoirs via platinum electrodes in the reservoirs. A function generator (33120A, Hewlett Packard, Palo Alto, CA, USA), in combination with a high-voltage amplifier (Bipolar Operational Power Supply/Amplifier BOP 1000M, Kepco, Flushing, NY, USA), or a high-frequency amplifier (WMA-300, Falco Systems, Amsterdam, The Netherlands) were used to provide the required signals. The voltage was confirmed with an oscilloscope (54603B 60 MHz, Hewlett Packard, Palo Alto, CA, USA) via a 1x/10x probe (Kenwood PC-54, 600 Vpp, Havant, UK). The experimental image stacks were captured with a monochrome Andor Neo sCMOS camera (Andor Technology, Belfast, Northern Ireland) or a color camera (Exmor USB 3.0, USB29 UXG M, Sony, Tokyo, Japan). The stacks were then analyzed using FIJI (ImageJ 1.52f, National Institutes of Health, Bethesda, MD, USA) and MATLAB (MathWorks, Natick, MA, USA). To measure the conductivity of the media, we used a conductivity meter (B-771 LAQUAtwin, Horiba Instruments, Kyoto, Japan). The zeta potentials of cells were measured with a Zetasizer NanoZS instrument (Malvern Instruments, Ltd., Worcestershire, UK).

### 2.2. Data Analysis

For both *S. cerevisiae* and *E. coli*, sorting performance during an experiment is assessed by comparison of lateral positions of cell populations at the beginning and at the end of the DLD array. The degree of displacement is given as the number of gaps from one side to the other at the end of the device. Particles that follow the flow exit at low gap numbers, and particles that are displaced exit at high gap numbers. However, when plotting the results, we convert the gap numbers to percentages (0%: no displacement, 100%: maximal displacement) as this makes it easier to compare results from devices with different numbers of gaps. The particles are counted in two ways. Manual counting, cell by cell, is accurate but can be labor intensive. This method was performed for the yeast cells, which are nonfluorescent. For the cells that are fluorescent (viable/non-viable *E. coli*), the fluorescence intensity is used as well to deduce the numbers of particles, which we will refer to in the plots as inferred counts. More details of the image processing can be found in Figure S1 of the Supplementary Information.

In the experiments with *E. coli* where the bacteria were sorted into different outlet reservoirs, the numbers of viable and non-viable cells recovered from the sample and the outlet reservoirs were evaluated. First, the recovered suspension from each outlet reservoir was pipetted into a centrifuge tube and concentrated by centrifugation. After that, the concentrated suspension was pipetted on a microscope slide, covered, and sealed with a cover slip. The cells were then imaged and counted to give the ratio of viable to non-viable cells.

### 2.3. Sample Preparation

We use bacteria and yeast cells as model systems to demonstrate proof of principle of our devices. Key information about the cells can be found in Section 3 of the Supplementary Information.

Green fluorescent *Escherichia coli* (2566/pGFP) (approximately 1.5 µm × 3 µm) were cultured and stored at −80 °C in culture medium with 20% *w*/*v* glycerol. Prior to experiments, the bacteria were allowed to thaw at room temperature. The concentration of the bacteria was measured at 8.2 × 10^8^/mL, using a DMS cell density meter with 600 nm light (DMS-cuvette, LAXCO Inc., Bothell, WA, USA). The bacteria were then spun down and suspended in running medium (KCl + 0.1% *w*/*v* Pluronic^®^ F127, σ = 20, 100, or 500 mS/m) at the same concentration. Half of the sample was kept at 70 °C for 20 min to kill the cells and then both halves were stained with propidium iodide (PI) (Sigma-Aldrich Sweden AB, Stockholm, Sweden) at a concentration of 20 µg/mL for 5 min. Propidium iodide is a dead-cell stain, which can penetrate compromised cell membranes. Viable *E. coli* bacteria will appear green due to the GFP, prior to and after PI staining. The heat-treated *E. coli* bacteria will appear dark prior to PI staining and orange after PI staining (using our microscope setup), enabling viable and non-viable heat-treated cells to be distinguished. The staining revealed that, prior to running experiments, in the “viable” population, around 80–90% of the cells were actually viable and in the heat-treated “non-viable” population, around 90–95% were actually non-viable.

Baker’s yeast cells (*Saccharomyces cerevisiae*) (*D* ~ 4.5 µm) in dry form (Jästbolaget AB, Sollentuna, Sweden) were suspended in glucose 5% *w*/*v* and heated up to 32 °C in a well-ventilated tube for 30 min, for activation. Half of the sample was then heat treated at 62 °C for 15 min to kill the cells. The viable and heat-treated samples were mixed at a ratio of 1:1 and stained with Trypan Blue at a concentration of 0.2% *w*/*v*, for 5 min. Trypan Blue is a common dead-cell stain, which permeates compromised cell membranes, leaving the non-viable cells dark blue and enabling viable and non-viable cells to be distinguished from one another. The mixed sample was then washed several times with the running media, consisting of KCl at different conductivities and 0.1% *w*/*v* Pluronic^®^ F127. The staining revealed that, prior to running experiments, in the “viable” population, around 70% of the cells were actually viable and in the heat-treated “non-viable” population, more than 90% were actually non-viable

## 3. Results and Discussion

We first characterize the different cell types with respect to size and zeta potential. See Table S2 in the Supplementary Information. Different experimental parameters are then explored by testing devices with different critical sizes, and different combinations of applied pressure, applied voltage, frequency of the applied voltage and conductivity of the buffer. Finally, we show that we can sort the *E. coli* with respect to zeta potential and, by selecting slightly different experimental conditions, the yeast based on what we believe is DEP. Estimated throughputs and Péclet numbers are given in Table S3 of the Supplementary Information.

### 3.1. Sorting of Viable/Non-Viable E. coli

We measured the size of rod-shaped *E. coli* (~2.5 µm × 1.5 µm, Figure S2, Supplementary Information) and found that the size differences between viable and non-viable bacteria are negligible. It is, therefore, not possible to separate them by size. We could, however, measure a significant change in the zeta potential (approximately −42 and −34 mV for viable and non-viable bacteria, respectively) using the Zetasizer™.

To find optimal experimental conditions for sorting the two different cell states, we started by selecting a suitable device. Sorting device #2 (*D_C_* = 0.64 µm) was found to have a too small critical diameter to function (Figure S3, Supplementary Information). Viable and non-viable cells had overlapping distributions at the end of the device and both were in displacement mode even when no voltage was applied. With an applied voltage at 1 to 10 Hz, displacement increased and overlap remained.

We, therefore, shifted to Sorting device #1 (*D_C_* = 1.24 µm), where both viable and non-viable cells travelled in zigzag mode when no voltage was applied. Supplementary Information Video S1 and Video S2, show bacteria distributions at the inlet and at the outlet, respectively. As the applied AC voltage was increased from zero, the cells transit from zigzag to displacement mode, and the viable cells tended to displace more easily than the non-viable cells, leading to separation (Supplementary Information Video S3).

**Figure 4:**
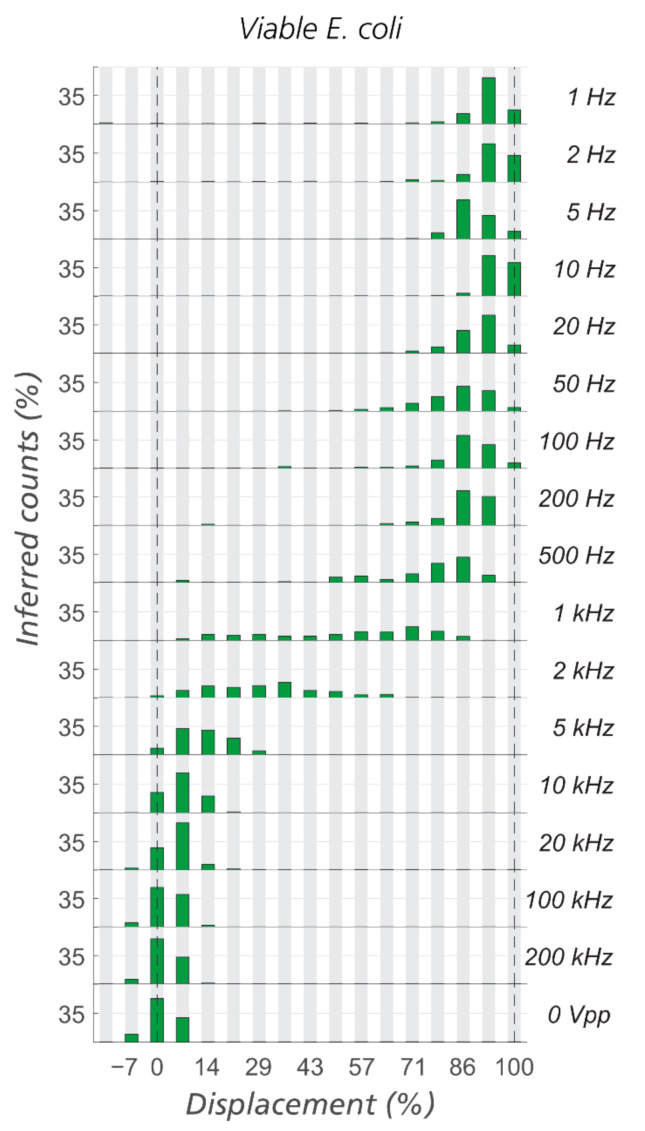
Effect of frequency on displacement of viable *E. coli* in eDLD. Analytical device #1 with gap 4 µm, *N* = 10, *D_C_* = 1.85 µm, medium conductivity = 25 mS/m, *V* = 300 V_PP_, and Δ*P* = 9.5 mBar.

The first test now is to scan the frequency from single Hz to hundreds of kHz. Figure 4 demonstrates clearly that viable *E. coli* are not displaced for frequencies above 1 kHz indicating that their zeta potential will determine their trajectories through the device rather than their dielectrophoretic mobility [20,35].

**Figure 5:**
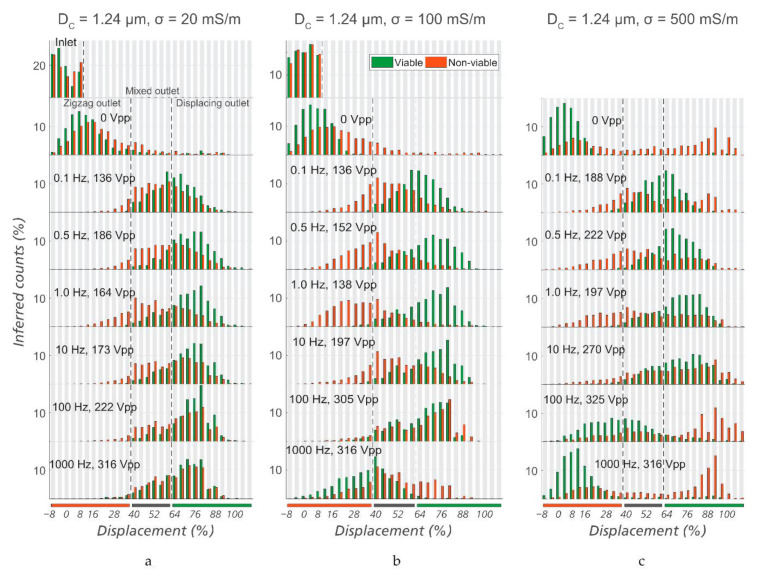
Optimization of sorting conditions. Lateral displacement of viable and non-viable *E. coli* at the outlet of the device (Sorting Device #1, *D_C_* = 1.24 µm), at different frequencies, voltages, and conductivities of the media: (**a**) Medium conductivity of 20 mS/m, (**b**) medium conductivity of 100 mS/m, and (**c**) medium conductivity of 500 mS/m. It can be seen that in general, *σ* = 100 mS/m gives the best separation among the three. Specifically, the optimized conditions for sorting are *σ* = 100 mS/m, *f* = 1.0 Hz, and *V* = 138 V_PP_.

Since the zeta potential is an important sorting parameter, we expect that it is necessary to carefully select buffer conductivity for optimal sorting. Therefore, for three different conductivities, we varied the frequency of the applied electric field in order to find an optimal combination of these two parameters. See Figure 5 for a summary of the data. The given voltages represent the lowest values for which a clear separation could be observed.

As the frequency is increased from 1 Hz, a higher voltage is needed to steer viable cells into the “displacing reservoir” (denoted by the green bar under the plots) and the “intermediate reservoir” (denoted by the grey bar). At 1 kHz, even a voltage of more than 300 V_PP_ (the limit of our amplifier) could not fully displace viable bacteria. It can be seen that 1 Hz was the best frequency for sorting, at a conductivity of 100 mS/m. At conductivities of 20 or 500 mS/m, similar trends were observed but the sorting capability was less efficient than at 100 mS/m. This is consistent with what we see for polystyrene microspheres as well as lipid vesicles in [20]. The increase in zeta potential of the particles due to the lower ionic strength contributes to the improved sorting.

Note the appearance of large aggregates at high conductivities (Figure 5c). We believe that the screening of the particle charge at the high conductivity of 500 mS/m gives rise to the aggregation. Supplementary Information Video S4 and Video S5, show viable and non-viable *E. coli* suspended in KCl at 0.5 mS/m and 500 mS/m, respectively, and support this view. While almost all non-viable *E. coli* (orange) in 0.5 mS/m medium are singles, many of the non-viable bacteria in 500 mS/m medium form large aggregates.

In the end, the optimized combination of conditions for sorting viable and non-viable *E. coli* was found to be a medium conductivity of 100 mS/m, a sinusoidal voltage of 1 Hz/138 V_PP_ in Sorting device #1 (*D_C_* = 1.24 µm) at an applied pressure of 20 mBar.

**Figure 6:**
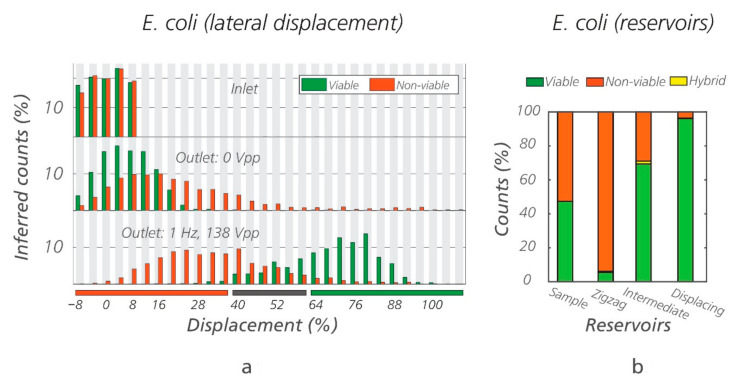
Sorting of viable/non-viable *E. coli* using electrokinetic DLD (eDLD). (**a**) Lateral displacement of the viable and non-viable *E. coli* based on fluorescence intensity at the beginning and at end of a DLD array (Sorting device #1, *D_C_* = 1.24 µm), with and without an applied field. The displacement corresponding to the zigzag/intermediate/displacing reservoirs have been marked with the orange/gray/green horizontal bars, respectively. Note that there seems to be some separation of non-viable cells from viable cells even when the electric field is switched off, probably due to the higher probability of non-viable cells to form aggregates. While roughly half of the non-viable cells can be collected, the remainder is still mixed with viable cells. For the general case, this level of sorting is of limited value. (**b**) External, manual counts of the ratio between viable and non-viable *E. coli* recovered from different outlet reservoirs. “Hybrid” refers to the few cells emitting both green and orange color, which represents a minute subset of the counted cells.

Using the optimal conditions, a mixture of viable/non-viable *E. coli* in equal proportions was run through the eDLD device (Sorting device #1, *D_C_* = 1.24 µm) for 1 h and 30 min. After sorting, the populations of the bacteria in the three outlet reservoirs were recovered and counted externally. The outlet reservoirs are named “zigzag, intermediate, and displacement,” corresponding to the trajectories of the particles they collect. The results for one such experiment are shown in Figure 6, demonstrating high purity of non-viable cells in the “zigzag reservoir” and high purity of viable cells in the “displacing reservoir.” To illustrate the reproducibility of results, three additional repetitions were carried out (Figure S4 of the Supplementary Information). We estimated, based on fluorescent intensity (Figure 6a), that 72% of the viable bacteria were recovered in the displacing reservoir, with a purity, based on manual count (Figure 6b), of more than 90%. Likewise, 63% of the non-viable bacteria were recovered in the zigzag reservoir, with a purity of more than 90%. The remaining cells exit at the intermediate reservoir. Note that depending on the application, purity could be increased at the expense of decreased recovery rate or vice versa by varying the design of the device, more specifically the placement of the exit channels and collection reservoirs. In Section 4 of the Supplementary Information, we show a full range of possible purity and recovery rates when varying placements of the zigzag and displacing reservoirs, see Figure S6.

### 3.2. Sorting of Viable/Non-Viable Yeast Cells

We measured the size of yeast cells (~2.5 µm × 1.5 µm), Figure S5, and found that the size differences between viable and non-viable bacteria is greater than for *E. coli*, but still small relative to the width of the size distributions of both types (non-viable: 3.80 ± 0.44 µm and viable: 4.70 ± 0.63 µm). In contrast to *E. coli*, the measured zeta potential of yeast cells was observed to change very little due to heat inactivation and was not expected to be useful for separation of the two subpopulations (~−19.5 mV, Table S2, Supplementary Information).

We measured the size of yeast cells (~2.5 µm × 1.5 µm), Figure S5, and found that the size differences between viable and non-viable bacteria is greater than for *E. coli*, but still small relative to the width of the size distributions of both types (non-viable: 3.80 ± 0.44 µm and viable: 4.70 ± 0.63 µm). In contrast to *E. coli*, the measured zeta potential of yeast cells was observed to change very little due to heat inactivation and was not expected to be useful for separation of the two subpopulations (~−19.5 mV, Table S2, Supplementary Information).

**Figure 7:**
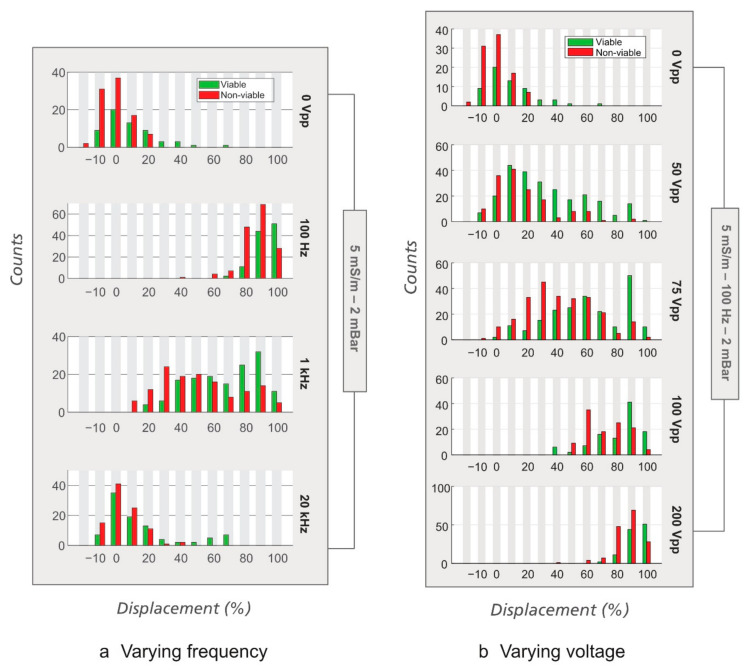
Effect of frequency and voltage on displacement of yeast cells at the outlet of the device. Analytical device #4 (*D_C_* = 5.6 µm) was used with *σ* = 5 mS/m and ∆*P* = 2 mBar. (**a**) Varying frequency. Note the trend that the displacement increases equally for both cell types as the frequency is decreased. The applied voltage was 300 V_PP_, except at 100 Hz, which is 200 V_PP_. (**b**) Varying voltage. The frequency was kept at 100 Hz.

We first tested the effects of the frequency and the applied voltage. When changing frequency (Figure 7a), we observed that at low frequency (~100 Hz), yeast cells experience stronger displacement than at higher frequency (1 and 20 kHz), just like polystyrene beads [20] and *E. coli*. When the changing parameter was voltage while the frequency was kept at 100 Hz (Figure 7b), both the viable and the non-viable yeast cells were displaced quite strongly and the displacement monotonically increases with the applied voltage. Separation was, however, not observed at any voltage due to the lack of contrast in electrical properties (zeta potentials) between the viable and the non-viable yeast cells.

**Figure 8:**
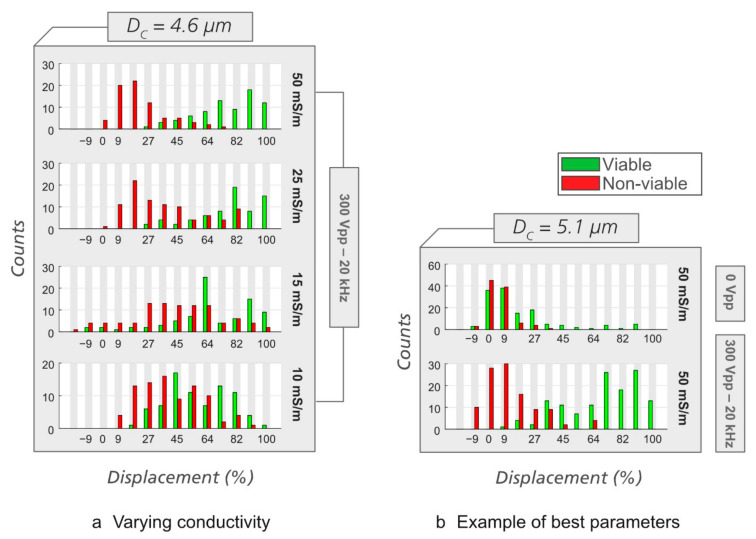
Displacement of viable/non-viable yeast cells at varying medium conductivities and in devices with different *D_C_*. (**a**) Varying conductivity. We use Analytical device #2, *D_C_* = 4.6 µm. In each experiment, the pressures were adjusted to maximize separation (from top to bottom, 1.5, 1.2, 0.7, and 0.6 mBar). (**b**) An example of a set of parameters that gives clear separation (Analytical device #3, *D_C_* = 5.10 µm, *σ* = 50 mS/m, *f* = 20 kHz, *V* = 300 V_PP_, ∆*P* = 1.2 mBar).

As in the case of *E. coli*, we then performed experiments at different medium conductivities and in different devices to find the best condition for sorting viable/non-viable yeast. It can be seen in Figure 8a that medium conductivity affects displacement of viable and non-viable yeast cells in eDLD. At the same applied voltage and frequency, some conductivity values give better separation between the viable and the non-viable cells than the others. We found the combination of *σ* = 50 mS/m, *V* = 300 V_PP_, *f* = 20 kHz, and ∆*P* = 1.5 mBar for Analytical device #2, *D_C_* = 4.60 µm, or ∆*P* = 1.2 mBar for Analytical device #3, *D_C_* = 5.10 µm, to be optimal for separation of viable/non-viable yeast cells. Such optimal separation is illustrated in Figure 8b. Supporting videos can be found in the Supplementary Information (Video S6: without the electric field and Video S7: with the electric field).

While the low-frequency approach was unsuccessful for yeast, by separating the yeast cells at higher frequency, we demonstrate one of the strengths of our method, namely, frequency can be used to target different types of morphological or structural changes in cells. Since electroosmosis and electrophoresis can be assumed to be negligible at 20 kHz, it is reasonable to assume that DEP makes an important contribution to the enhanced displacement and sorting in this case.

## 4. Conclusions and Future Work

We have presented and characterized an integrated device that combines DLD and electrokinetics to sort particles based not only on size but also on electric and dielectric properties. Since changes in viability are linked to changes in properties such as surface charge (zeta potential), membrane integrity, membrane conductivity, and polarizability rather than size or shape, we were able to show proof of principle of leveraging these parameters to perform viability-based separations.

We note that the *E. coli* and yeast separations require different ranges of frequency to function. This implies the existence of at least two different mechanisms that make eDLD work: one at low frequency, related to electroosmotic flow (EOF) and electrophoresis (EP) (as we discuss in [20]), and the other at higher frequency, related to DEP. At this point we do not know the exact mechanisms. We can rule out DEP as the main mechanism for the low-frequency cases as well as most probably linear EOF and EP. Instead, we posit that non-linear EOF and EP may play an important role [20]. However, further studies are necessary to fully understand how these mechanisms interact and are responsible for the separation. Nevertheless, we have demonstrated that we can select different underlying mechanisms and optimize running conditions such as ionic strength, applied voltage and pressure, and the geometry of the device, to adapt the approach to two different cells with different physical characteristics that in turn are coupled to relevant biological subpopulations.

Potential applications that we envision are applications in food industry to monitor the effect of the viability of any microorganisms used in the processing of the food and those microorganisms that are not desired. From a fabrication perspective, the design is simple and can be realized in cheap materials using standard mass production techniques.

## Figures and Tables

**Table 1:** Analytical deterministic lateral displacement (DLD) devices. Critical diameters, *D_C_*, are nominal values given based on the geometry of the devices and Equation (1). Channel lengths are 4.25 ± 0.25 mm. The deflection width is approximately 425 µm corresponding to 85% of the device width.

Device Name	Gap (µm)	*N*	*D_C_* (µm)	Deflection, *θ*	Cell Type
**Analytical device #1**	4	10	1.9	5.71°	*E. coli*
**Analytical device #2**	10	10	4.6	5.71°	Yeast
**Analytical device #3**	11	10	5.1	5.71°	Yeast
**Analytical device #4**	12	10	5.6	5.71°	Yeast

**Table 2:** Sorting devices used for *E. coli*. The trajectories for the large particles (displacement mode) are illustrated through four different numbers: critical diameter, deflection angle, absolute deflection, and relative deflection. Critical diameters, *D_C_,* are nominal values given based on the geometry of the devices and Equation (1).

Device Name	Gap (µm)	*N*	*D_C_* (µm)	Channel Length (mm)	Deflection *θ*	Deflection (µm)	Deflection/Channel Width
**Sorting device #1**	4	23	1.24	9.5	2.49°	400	86%
**Sorting device #2**	3	50	0.64	22.9	1.15°	450	86%

## Data Availability

Essential data are contained within the article and the supplementary material. The raw data are available on request from the corresponding author.

## Supplementary Materials

The following are available online at https://www.mdpi.com/2072-666X/12/1/30/s1. Figure S1: Image processing steps, Figure S2: Dimensions of viable and non-viable *E. coli*, Figure S3: Displacement of viable and non-viable *E. coli* in *D_C_* = 0.64 µm device, Figure S4: Sorting of viable and non-viable *E. coli* in *D_C_* = 1.24 µm device, Figure S5: Dimensions of viable and non-viable yeast cells, Figure S6: Purity and recovery rates of viable and non-viable *E. coli*, Table S1: Device fabrication steps, Table S2: Properties of the cells, Table S3: Throughput and Péclet numbers of various experiments reported in the main text, Video S1: *E. Coli*—Sorting—100 mS_m—20 mBar—Inlet. Viable (green) and non-viable (orange) *E. coli* bacteria entering a DLD array having *D_C_* = 1.24 μm. The video was recorded in real time using the color camera. In the same experiment, the monochrome camera was used to obtain the videos from which the results were processed and plotted using the method described in Figure S1. The results are shown in the top plot of Figure 6a. Video S2: *E. coli*—Sorting—100 mS_m—20 mBar—Outlet. Same experiment as in Video S1, now captured at the outlet. No voltage was applied. The results are shown in the middle plot of Figure 6a. Video S3: *E. coli*—Sorting—100 mS_m—20 mBar—1 Hz—138 Vpp—Outlet. Continuing from Video 2, now with the AC voltage on. Separation of viable and non-viable cells is demonstrated. The results are shown in the bottom plot of Figure 6a. Video S4: *E. coli*—0d5 mS_m. At low medium conductivity, most of the non-viable *E. coli* (orange) stay single. Video S5: *E. coli*—500 mS_m. At high medium conductivity, most of the non-viable *E.* coli (orange) form clusters. Video S6: Yeast—Sorting—50 mS_m—0 Vpp—1d2 mBar. Viable (transparent) and non-viable (dark) yeast cells exiting at the end of a DLD array having *D_C_* = 5.1 μm. Both viable and non-viable cells were in zigzag mode and stay closer to the left wall. The video was cut out from the original video used to produce Figure 8b (0 V_PP_) and sped up two times. The original video is 10 times longer. Video S7: Yeast—Sorting—50 mS_m—20 kHz—300 Vpp—1d2 mBar. Similar to Video S6, but an AC voltage of 20 kHz, 300 V_PP_ was applied. While the non-viable cells were still in zigzag mode, the viable ones were displaced and appear closer to the right wall. The video was cut out from the original video used to produce the graph of Figure 8b (300 V_PP_) and sped up two times. The original video is 7.5 times longer.
