# Supplementary material for: Cell Sorting Using Electrokinetic Deterministic Lateral Displacement
Source: Micromachines (Basel). 2020 Dec 30;12(1):30. doi: 10.3390/mi12010030 (PMC7823954; doi:10.3390/mi12010030)
Supplement: Supplementary file 1 [file micromachines-12-00030-s001.zip › Adjusted ESI files_zipped/ESI_ver7.docx]

Supplementary Information

Cell Sorting using Electrokinetic Deterministic Lateral Displacement

Bao D. Ho ^1^, Jason P. Beech ^1^ and Jonas O. Tegenfeldt ^1,^*

^1^ Division of Solid State Physics and NanoLund, Physics Department, Lund University, PO Box 118, 22100, Lund, Sweden; [bao.hodang@gmail.com](mailto:bao.hodang@gmail.com) (B.D.H.); [jason.beech@ftf.lth.se](mailto:jason.beech@ftf.lth.se) (J.P.B.)

***** Correspondence: [jonas.tegenfeldt@ftf.lth.se](mailto:jonas.tegenfeldt@ftf.lth.se); Tel.: +46 46 222 8063

1. Device fabrication

**Table S1.** Device fabrication steps.

| **Tasks** | **Steps** | **Vendors/Reference** |
| --- | --- | --- |
| Making SU-8 mold | 1. Design a photo mask using L-Edit 2016 | L-Edit 2016: Mentor Graphics Corp., Wilsonville, Oregon, United States |
|  | 1. Have the mask printed by a mask supplier | Delta Mask, Enschede, The Netherlands |
|  | 1. Dispense SU-8 onto a 3-inch silicon wafer and spin at a well-defined speed, according to the manufacturer’s instructions. This defines the thickness of the SU-8 layer and consequently the depth of the molded PDMS device. | SU-8: MicroChem, Newton, MA, USA |
|  | 1. Expose the wafer to UV-light in a Karl Suss contact mask aligner, then develop the un-exposed SU-8. | Karl Suss MJB4 soft UV, Munich, Germany |
|  | 1. Coat the SU-8 mold with a monolayer of trichloro (1H,1H,2H,2H-perfluorooctyl) silane (PFOTS) by molecular vapor deposition to prevent PDMS from sticking to the mold over many tens of uses | PFOTS: Sigma Aldrich, Saint Louis, MO, USA |
| Making PDMS devices | 1. Mix PDMS base and PDMS curing agent (ratio 10:1 w/w) and de-gas to remove air bubbles | PDMS: Sylgard 184, Dow Corning, Midland, MI, USA |
|  | 1. Pour PDMS onto the SU-8 mold, and cure in an oven at 80° C for 1 hour. |  |
|  | 1. Peel the PDMS slab off the mold, cut out the region containing the device, perforate the device at reservoir positions with a puncher (1 mm inner diameter) |  |
|  | 1. Treat the feature surface of the device and a PDMS-covered glass slide with oxygen plasma, and gently place the device on top of the glass slide. | ZEPTO, Diener Plasma-Surface Technology, Ebhausen, Germany |
|  | 1. Glue silicone tubes (5 mm outer, 3 mm inner) onto the device to form inlet and outlet reservoirs | Silicone tubes: 228-0707, VWR International LLC, Radnor, PA, USA  Glue: Elastosil A07, Wacker Chemie AG, Munich, Germany |
|  | 1. Cure the bonded devices in an oven at 120° C for 24 hours to bring the PDMS surface back to its native hydrophobic state, making it stable for electrokinetic experiments. Store the device for future use. |  |

2. Image processing

It is impossible to manually count individual moving *E. coli* cells, as can be done with yeast cells, since we ran the cells at high concentration in order to collect sufficient numbers in each outlet reservoir for further external staining and counting steps. Instead, the average fluorescence intensity of the cells from an image stack capture at the end of a DLD array can be utilized as a means to estimate the relative counts. The detailed image processing steps are illustrated in Figure S1, where steps from 1 to 4 are performed in ImageJ, and step 5 and 6 are done in MATLAB.


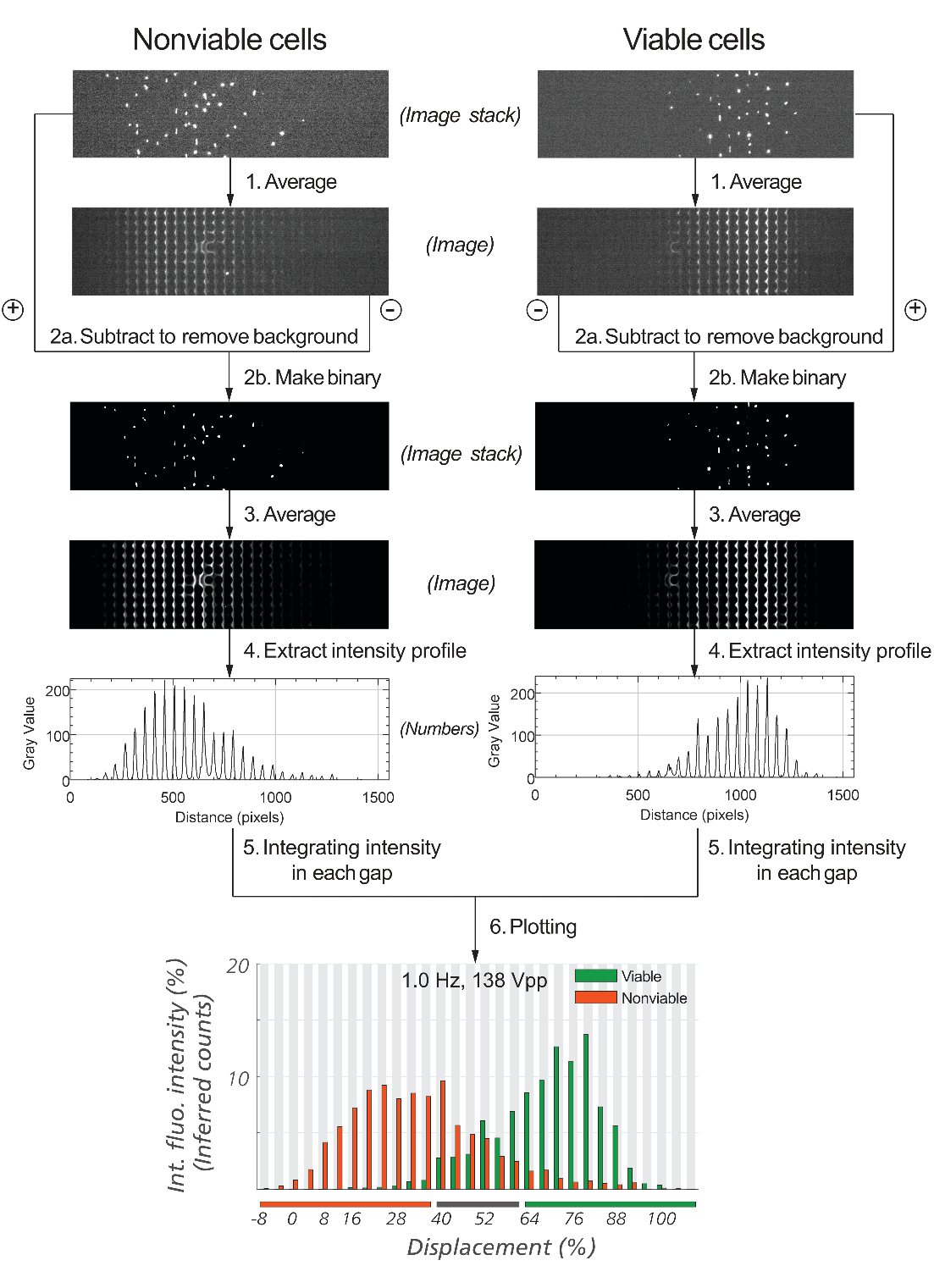


**Figure S1.** Flow chart of the detailed image processing and data extraction steps used to estimate relative cell counts based on fluorescence intensity. (Step 1) Taking an average of all images in the stack. (Step 2) Removing background (by subtracting the average intensity of the whole stack from each image in the stack) and making the images binary to remove speckles and noises. (Step 3) Averaging over all binary frames from step 2. (Step 4) Extract intensity profile. (Step 5) Normalize and bin the intensity across each gap. (Step 6) Plotting.

3. Cells used for the experiments

The properties of the cells used in our work are listed in Table S2. Sizes were measured by visual inspection using light microscopy. Medium conductivity was measured using the conductivity meter, B771 LAQUAtwin. Zeta-potential was measured using the Zetasizer. Concentration of the *E. coli* cells was measured with a DMS-cuvette (see the main text). Concentration of yeast cells was estimated based on flow rates and the numbers of cells passing through the devices.

**Table S2**. Specifications for the cells used in this work. The media are KCl solutions with optionally added Pluronic® F127 0.1 % w/v to prevent sticking. The concentration of KCl was adjusted so that the conductivity reached a desired value: KCl 50 mS/m (3.5 mM), KCl 100 mS/m (7.1 mM). These are the conductivities used in the experiments yielding the best separations for *E. coli* (100 mS/m) and yeast (50 mS/m).

| Cell types | Size (µm) | Medium conductivity (mS/m) | ζ-potential (mV) | Strain/Vendor | Fluorescence color/  Apparent color | Concentration | Main text subsection |
| --- | --- | --- | --- | --- | --- | --- | --- |
| *E. coli* (viable),  GFP | 2.83 ± 0.60 | 100 | -43.2 ± 6.2 | Strain 2566/pGFP | Green | 8.2×10^8^/mL | 3.1 |
|  | 1.48 ± 0.09 |  |  |  |  |  |  |
| *E. coli* (non-viable),  Propidium Iodide | 2.42 ± 0.48 | 100 | -34.1 ± 4.3 | Strain 2566/pGFP | Orange | 8.2×10^8^/mL | 3.1 |
|  | 1.44 ± 0.10 |  |  |  |  |  |  |
| *S. cerevisiae*  (viable) | 5.37 ± 0.72 | 50 | -19.5 ± 3.8 | Jästbolaget AB | Transparent  (Yellow-ish) | 0.6×10^6^/mL | 3.2 |
|  | 4.70 ± 0.63 |  |  |  |  |  |  |
| *S. cerevisiae*  (non-viable) | 4.49 ± 0.57 | 50 | -19.6 ± 3.5 | Jästbolaget AB | Opaque dark blue | 0.5×10^6^/mL | 3.2 |
|  | 3.80 ± 0.44 |  |  |  |  |  |  |

3.1. Bacteria (E. coli)

3.1.1. Dimensions of viable and non-viable E. coli

Viable and non-viable *E. coli* were imaged using an Exmor USB 3.0 color camera (see main text).


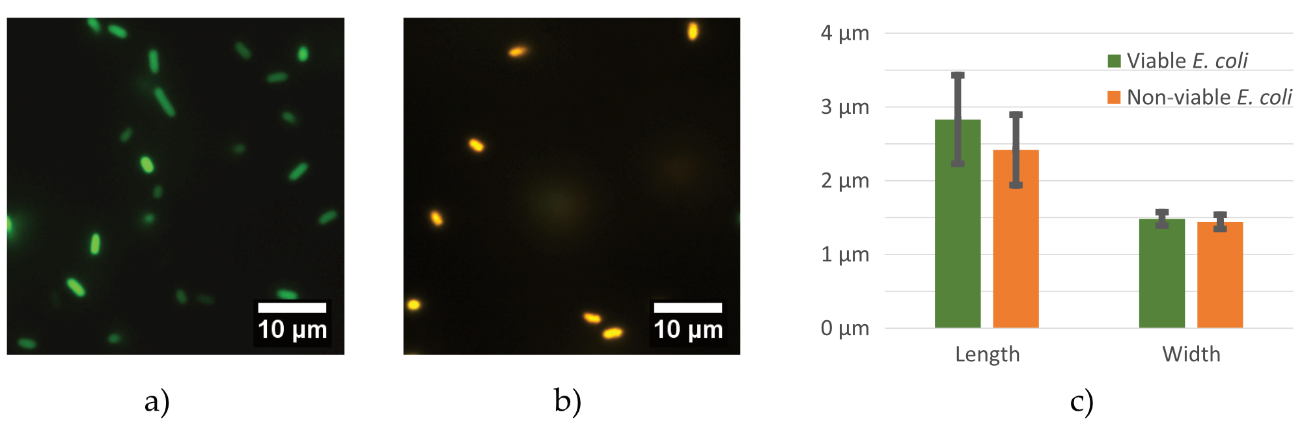


**Figure S2.** Viable and non-viable *E. coli.* a) Viable *E. coli* visualized by GFP fluorescence. b) Heat treated non-viable *E. coli*, stained with Propidium Iodide. c) Dimensions of *E. coli*, averaged over 61 cells (viable) and 46 cells (non-viable). The error bars show one standard deviation. Cell length (µm): non-viable: 2.42 ± 0.48, viable: 2.83 ± 0.60; cell width (µm): non-viable: 1.44 ± 0.10, viable: 1.48 ± 0.09.

3.1.2. Additional measurements

For a device with *D_C_* = 0.64 µm (Sorting device #2), both cell types were displaced even without any applied voltage (Figure S3). At 10 Hz and 316 V_PP_ both cell types were fully displaced and overlapped.


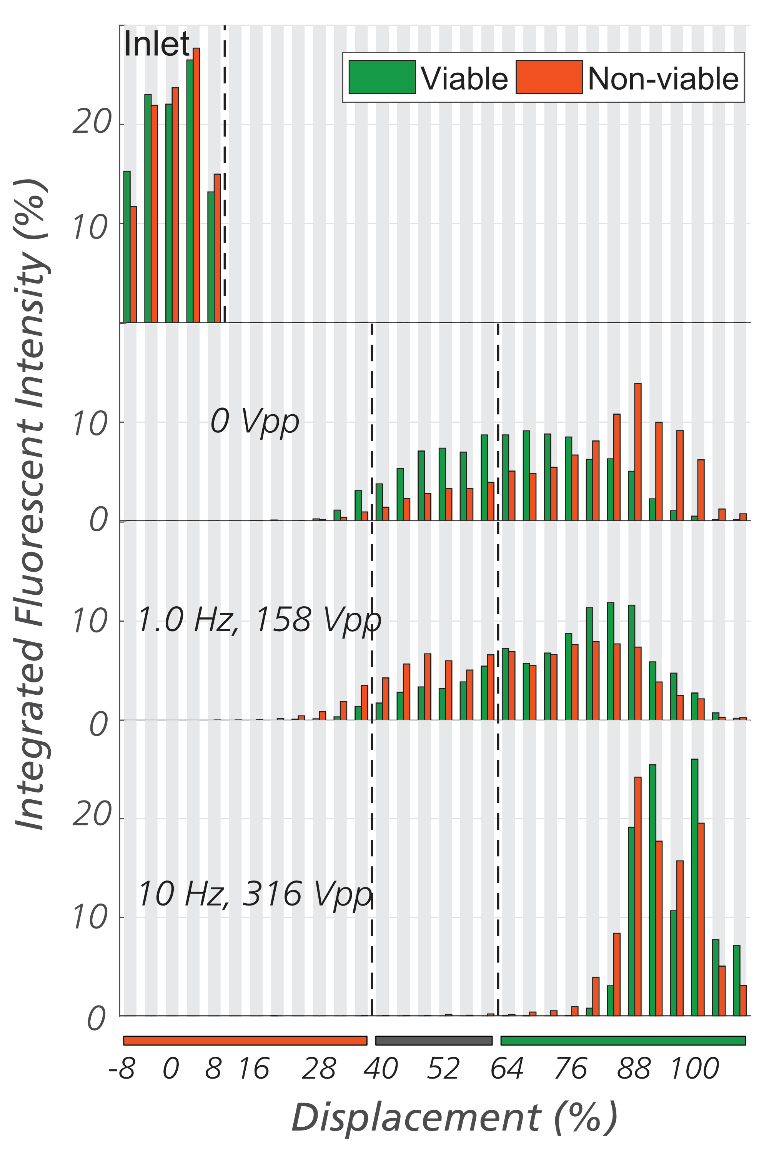


**Figure S3.** Lateral position of viable and non-viable *E. coli* at the outlet of the *D_C_* = 0.64 µm (Sorting device #2). Both viable and non-viable cells were displaced even when no AC field was applied, *D_C_* is too small. The conductivity was 100 mS/m.

To illustrate the variation in sorting results for the *E. coli*, the experiments took place in total four times. Figure S4 shows how viable/non-viable *E. coli* are sorted into different outlet reservoirs. The top view of the DLD device is shown in Figure S4a, with names of all inlet and outlet reservoirs. Figure S4b shows snapshots at the beginning and at the end of the DLD array during an experiment. Figure S4c supplements Figure 6b of the main text. The *E. coli* sorting experiments were run three times with viable/non-viable *E. coli* cell stained with PI before sorting and one time with the bacteria stained with PI only after sorting.


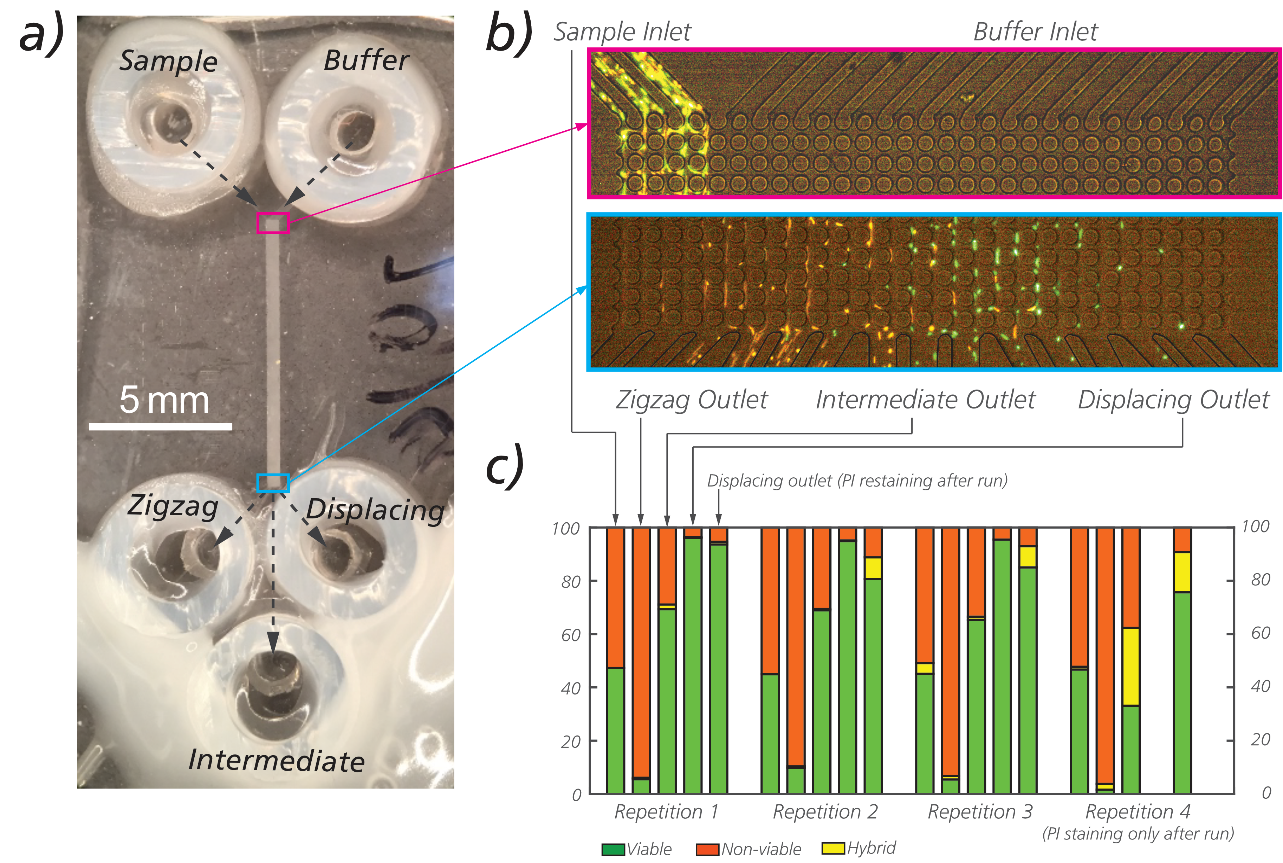


**Figure S4.** Sorting of viable/non-viable *E. coli* into different outlet reservoirs. a) Top view of the device. b) Snapshots of the cells at the beginning and at the end of the DLD array during an experiment. c) All repetitions of *E. coli* viable/non-viable cell sorting experiments. For repetition #1, #2, and #3, the samples were stained with Propidium Iodide before the experiments to aid visualization during the experiments. The sorted populations in three different outlet reservoirs were recovered and counted externally. To test for viability after sorting, the recovered populations in the displacing reservoir were stained again with PI and counted. For repetition #4, the sample was not stained with PI prior to the run, to confirm that the contrast between viable and non-viable cells is intrinsic and not because of PI absorption. The sorted populations were recovered, stained with PI, and counted. Hybrid refers to cells emitting both green and orange fluorescence color.

3.2. Yeast (Saccharomyces Cerevisiae)

3.2.1. Dimensions of viable and non-viable yeast cells

Viable and non-viable yeast were imaged using an Exmor USB 3.0 color camera (see main text).


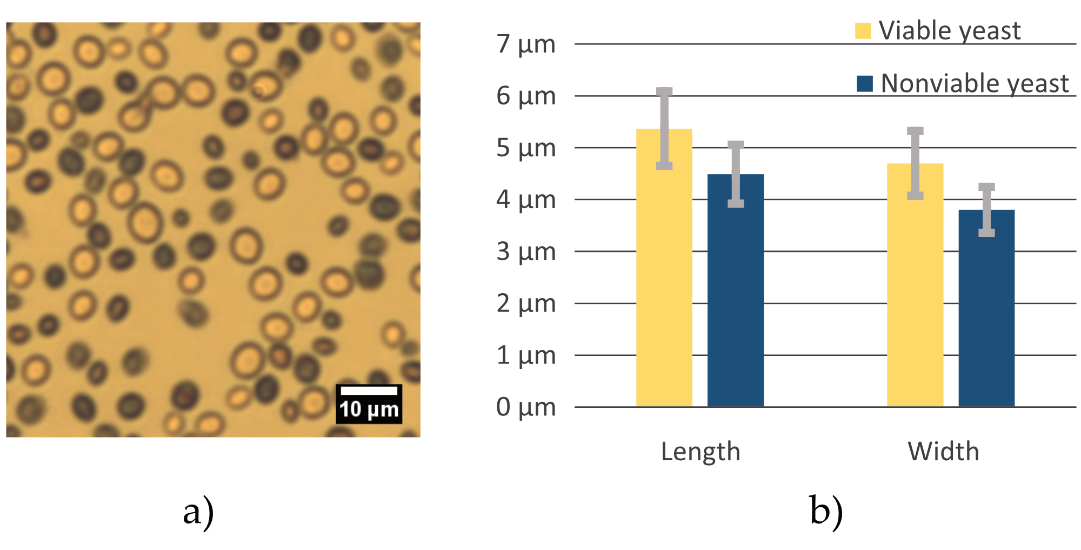


**Figure S5.** Viable and non-viable yeast cells. a) A mixture of viable yeast cells (transparent/yellow) and heat-treated non-viable yeast cells stained with Trypan Blue (dark blue). b) Dimensions of viable and non-viable yeast cells, averaged over 50 cells each. The error bars show one standard deviation. Cell length (µm): non-viable: 4.49 ± 0.57, viable: 5.37 ± 0.72; cell width (µm): non-viable: 3.80 ± 0.44, viable: 4.70 ± 0.63.

4. Optimizing purity and recovery

The end result of the sorting of the viable and non-viable cells is a balance between purity and recovery of the sample. Here we discuss how one can adjust the balance by simple changes in the design and layout of the devices with examples from the sorting of the bacteria.

In Figure 6a of the main text, we place the zigzag reservoir at the first 12 gaps, the intermediate reservoir at the next 6 gaps, and the displacing reservoir at the last 12 gaps. To put the experimental results in perspective, we first simplify by removing the intermediate reservoir, *i.e.* we consider a device with only two reservoirs. We then vary the relative width of the zigzag reservoir and calculate the corresponding purity and recovery rates of the viable and non-viable cells. Since the viable cells are larger, we aim to sort them into the displacing reservoir and the non-viable cells into the zigzag reservoir. We define purity and recovery rates as follows:

$$Purity \left( viable \right)=\frac{\#viable cells in dis. reserv.}{\#viable cells in dis. reserv.+\#nonviable cells in dis. reserv.}\times100$$

$$Purity \left( nonviable \right)=\frac{\#nonviable cells in zigzag reserv.}{\#nonviable cells in zigzag reserv.+\#viable cells in zigzag reserv.}\times100$$

$$Recovery \left( viable \right)=\frac{\#viable cells in dis. reserv.}{\#viable cells in dis. reserv.+\#viable cells in zigzag reserv.}\times100$$

$$Recovery \left( nonviable \right)=\frac{\#nonviable cells in zigzag reserv.}{\#nonviable cells in zigzag reserv.+\#nonviable cells in dis. reserv.}\times100$$

With the above definitions and using the distributions in the bottom plot of Figure 6a (main text), the purity and recovery can be plotted as a function of the width of the zigzag reservoir (Figure S6a). This plot can be used to design the zigzag and displacing outlets if a specific purity or recovery rate is required. Note that purity and recovery are both at 50% if no sorting ever happens. From Figure S6a, when the zigzag outlet width corresponds to the first 16 gaps, the purity and recovery rates for both types of cells are 86%.

As a comparison, we consider using a normal DLD for the same task of sorting viable/non-viable E. coli. In this hypothetical ideal case, we assume that we can vary *D_C_* to any value we want, that in the input sample the ratio between the numbers of viable and non-viable cells is 50/50, and that the width and length of the cells follow normal distributions with means and standard deviations stated in Figure S2. The purity and recovery can then be plotted as a function of *D_C_*, given the sorting parameter is the cell width (Figure S6b) or the cell length (Figure S6c). It can be seen that the sorting using an ideal non-electrokinetic DLD is poorer than with eDLD. For example, the purity of 86% can never be achieved for viable cells if sorting by width and for non-viable cells if sorting by length. To achieve 86% purity for non-viable cells if sorting by width, the recovery is only 2%. To achieve 86% purity for viable cells if sorting by length, the recovery is only 23%.


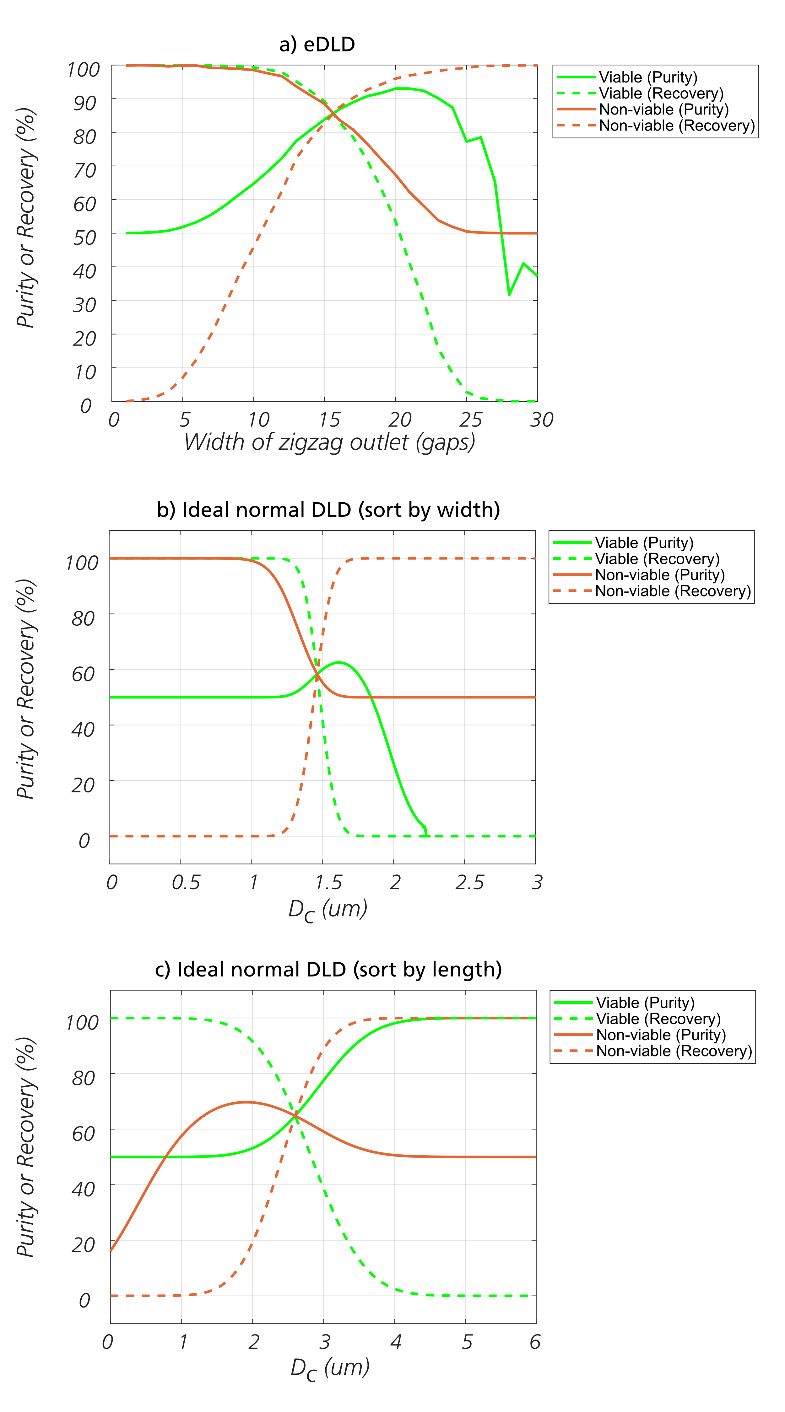


**Figure S6.** Purity and recovery rates of viable and non-viable cells in three different scenarios. a) In eDLD device, with the distribution plotted in Figure 6a of the main text. b) In an ideal normal DLD device, if width of the bacteria is the sorting parameter. c) In an ideal normal DLD device, if length of the bacteria is the sorting parameter.

5. Throughput and effect of diffusion

The throughputs of the devices are on the order of 1µL/hr and the Péclet numbers are much greater than unity. Below are specific calculations for Sorting device #1 that was used for the sorting of the viable/non-viable *E. coli*. Results are also given for Analytical device #3 at the end of the section.

At 20 mBar applied pressure difference across the device, the maximum velocity of the fluid is estimated at 300 µm/s (based on observed particle velocity). Assuming parabolic flow and noting that the depth (10µm) >> gap (4µm) between the posts, this corresponds to an average velocity of *v_avg_* = 2/3 *v_max_* = 200 µm/s.

With 30 gaps across the width of the DLD channel the volumetric flow rate is estimated at:

$$Q = 30\times A_{cross_{section}}\times v_{avg}$$

$$Q= 30\times4 \mu m\times10 \mu m\times200 \mu m/s= 240 000 \mu m^{3}/s$$

$$Q = 0.864\times{10}^{9} \mu m^{3}/hour \sim1 \mu L/hour$$

We estimate the Péclet number as follows.

$$Pe \# = X^{2} / \sigma^{2}$$

Here the advective length scale *X* is the deflection length of the displacing particle. This is the length scale of interest since separation breaks down if diffusion is significant when compared to this length. The deflection of particles in displacement mode in Sorting device #1: $X = 400 \mu m$.

The time it takes to transport particles the whole length of the DLD array is then simply:

$$t = L/v_{avg}=9500 \mu m / (200 \mu m/s) \approx48 s$$

Diffusion coefficient (assuming 1 µm sphere in water):

$D = kT/6\pi\eta a= 4\times{10}^{-21}J/(6\times3.14\times{10}^{-3}kg/(m.s)\times{0.5\times10}^{-6}m)$

$$D \approx4\times{10}^{-13} m^{2}/s = 4\times{10}^{-1} \mu m^{2}/s$$

Diffusion length:

$$\sigma^{2}= 2Dt = 2\times4\times{10}^{-1} \mu m^{2}/s* 48s$$

$$\sigma^{2}= 38 \mu m^{2}$$

This gives us a Péclet number of $Pe \approx4000$ for 1 µm particles in Sorting device #1.

Note that for these conditions, even with a 100 nm particle, the $Pe\approx400\gg1$.

To give an idea of how throughputs and Péclet numbers vary for the different samples in this work, results for different particles are shown in Table S3.

**Table S3**. Throughput and Péclet numbers of various experiments reported in the main text: E.coli (main text subsection 3.1) and Yeast (main text subsection 3.2).

| Cell type | Cell diameter, rounded (µm) | Device | D_C_ (µm) | Pressure (mBar) | v_avg_ (µm/s) | Throughput (µL/hour) | Peclet number |
| --- | --- | --- | --- | --- | --- | --- | --- |
| *E. coli* | 1.0 | Sorting device #1 | 1.24 | 20 | 200 | 0.86 | 3.9×10^3^ |
| *S. cerevisiae* | 4.0 | Analytical device #3 | 5.10 | 1.2 | 83 | 0.92 | 14.2×10^3^ |
